# Application of per-Residue Energy Decomposition to Design Peptide Inhibitors of PSD95 GK Domain

**DOI:** 10.3389/fmolb.2022.848353

**Published:** 2022-03-30

**Authors:** Miao Tian, Hongwei Li, Xiao Yan, Jing Gu, Pengfei Zheng, Sulan Luo, Dongting Zhangsun, Qiong Chen, Qin Ouyang

**Affiliations:** ^1^ Key Laboratory of Tropical Biological Resources of Ministry of Education, School of Pharmaceutical Sciences, Hainan University, Haikou, China; ^2^ Department of Pharmaceutical Chemistry, Third Military Medical University, Chongqing, China; ^3^ Department of Neurology, Xinqiao Hospital, Third Military Medical University, Chongqing, China

**Keywords:** PSD95 GK, peptides, molecular dynamics simulation, energy decomposition, MM-GBSA

## Abstract

Specific interaction between the postsynaptic density protein 95 (PSD95) and synapse-associated protein 90/postsynaptic density 95–associated protein (SAPAP) is crucial for excitatory synaptic development and plasticity. Designing inhibitors that target the guanylate kinase (GK) domain of PSD95, which is responsible for the interaction, is a promising manipulation tool for the investigation of the function of PSD95 GK and the etiology of its related psychiatric disorders. Herein, we designed new peptide inhibitors of PSD95 GK/SAPAP with higher binding affinity by using molecular dynamics simulations. First, the interactions between PSD95 GK and their reported phosphorylated and unphosphorylated peptides were explored by molecular dynamics simulations. Besides the hydrogen bonding interactions mediated by the phospho-serine (p-Ser) or corresponding phosphomimic residue Asp/Glu, the hydrophobic interactions from the other amino acids also contribute to the PSD95 GK/SAPAP interaction. As an unphosphorylated synthetic peptide with moderate binding affinity and relatively lower molecular weight, the QSF inhibitory peptide was selected for further modification. Based on per-residue energy decomposition results of the PSD95 GK/QSF complex, ten peptides were designed to enhance the binding interactions, especially the hydrophobic interactions. The top-ranked five peptides with lower binding energy were eventually synthesized. The binding affinities of the synthesized peptides were determined using fluorescence polarization (FP) assay. As expected, all peptides have higher binding affinity than the QSF peptide (*K*_i_ = 5.64 ± 0.51 μM). Among them, F10W was the most potent inhibitor (*K*_i_ = 0.75 ± 0.25 μM), suggesting that enhancement of the hydrophobic interactions is an important strategy for the design of new inhibitory peptides targeting PSD95 GK.

## Introduction

Psychiatric disorders such as autism spectrum disorder (ASD), obsessive compulsive disorder (OCD), intellectual disability (ID), and schizophrenia (SCZ) are a category of mental disorders caused by thousands of genetic variants (Rudan, 2010; Dwyer, 2020). About 20–25% of the population is suffering from such diseases, which have a large impact on cognitive impairment and become a major health issue in societies (Stein et al., 2010; Prasad and Satyanand, 2016). Postsynaptic density protein 95 (PSD95) belongs to the membrane-associated guanylate kinase (MAGUK) family of scaffolding proteins and mainly localizes at excitatory synapses, consisting of three PDZ domains, one SH3 domain, and one catalytically inactive GK domain (Montgomery et al., 2004; Funke et al., 2005). At synapses, it assembles the PSD95/SAPAP/shank complex and exerts a crucial role in synaptic formation and plasticity (Grant, 2012; Marín, 2012). Extensive genetic studies have shown that PSD95 is implicated in psychiatric disorders (Cline, 2005; Coley and Gao, 2018). For example, mutations of *DLG4* (encoding PSD95) are associated with both SCZ and autism, and a missense mutation of *DLG4* (T611I) that weakens the PSD95/SAPAP interaction could interfere with synapse development and cause ID (Hahn et al., 2006; Rauch et al., 2012; Hashimoto et al., 2013; Lelieveld et al., 2016). Therefore, developing peptides or small molecules that disturb the interactions of the PSD95/SAPAP is useful to investigate the function of PSD95 GK and determine the mechanism of the etiology of its related psychiatric disorders.

Previous studies have revealed that GK domains of the MAGUK family scaffold proteins evolved from the enzyme guanylate kinase which catalyzes the phosphorylation of GMP to GDP into phosphopeptide binding modules (Zhu et al., 2012). Although the GK domain has lost catalytic activity and shows very weak binding affinity to GMP, the GMP-binding site (the phosphor-site) of the GK domain serves as a crucial binding pocket which accommodates the specific p-Ser of the phosphor-peptide. The phosphorylation-dependent interactions are well-characterized in the previously reported GK domain’s partners such as p-LGN (a mitotic spindle regulatory protein), p-LGL2 (a mammalian homolog of *Drosophila* lethal giant larvae, Lgl), and p-SAPAP1 (also known as DLGAP1 or GKAP) (Zhu et al., 2011; Zhu et al., 2014; Zhu et al., 2017). The phosphate group of the phosphor-peptides extensively interacts with a number of residues from the phosphor-site of PSD95 GK (R568, Y580, and Y609). Exclusion of this phosphate would exclude the main binding energy, resulting in the significant loss of binding affinities (Zhu et al., 2011). However, phosphor-peptides are often unstable *in vivo* due to the easy degradation by enzymes and having low cell penetrability (Dunican and Doherty, 2001; Ye et al., 2007; Fuhs and Hunter, 2017).

Recently, the structure of PSD95 GK/MAP1A revealed the phosphorylation-independent mode of the GK domain, which could also be found in the complex of PSD95 GK/KIF13B MBS (Zhu et al., 2016; Xia et al., 2017). In the phosphorylation-independent binding mode, the residues Asp or Glu mimic the p-Ser in binding to the “phosphor-site” of PSD95 GK. Furthermore, the non–phosphor-peptides QSF, MKL, and DLS were developed which can effectively disrupt the PSD95 GK/SAPAP interaction (Zhu et al., 2017). Most recently, a series of stapled peptide inhibitors were also developed by molecular dynamics simulations with the best binding affinity (*K*_d_) of 1.36 μM using the strategy of increasing the stability of the stapled peptide in free solution (Unarta et al., 2021). However, the binding affinity of these non–phosphor-peptides is still limited and needs to be improved for further drug development.

Molecular dynamics (MD) simulation studies have successfully assisted the development of many novel peptides against various drug targets (Taylor and Jayaraman, 2020; Aronica et al., 2021; Damjanovic et al., 2021). MD simulations facilitate researchers to investigate the interactions of desired biological targets in terms of the binding energies at decreasing costs with advanced simulation methods (Veselovsky and Ivanov, 2003; Aminpour et al., 2019). Moreover, molecular mechanics generalized born surface area (MM-GBSA) methods coupled with per-residue energy decomposition studies become the commonly used approaches computing the binding free energies, which could elucidate the energy contribution of each amino acid and aid for the rational drug design of desired targets (Genheden and Ryde, 2015; Wichapong et al., 2021).

Herein, MD simulations and MM-GBSA methods were utilized to develop new potent non–phosphor-peptides of PSD95 GK. We investigated the hot residues at the interface and elaborated the key residue interactions of the complex by implementing the decomposition of binding free energy. According to the analysis of the interactions, a series of new peptides were then designed, synthesized, and evaluated using fluorescence polarization assay. Eventually, we have successfully developed new peptide inhibitors of PSD95 GK with 0.75 ± 0.25 μM of *K*_i_ value.

## Method

### Protein Preparation

The crystal structures of PSD95 GK and peptide binders were downloaded from the RCSB Protein Data Bank (PSD95 GK/p-LGL2a: 3WP0, PSD95 GK/p-LGL2b: 3WP1, PSD95 GK/p-SAPAP1: 5YPO, PSD95 GK/MAP1A: 5GNV, PSD95 GK/QSF: 5YPR). The water molecules in the complex were deleted, and then the missing amino acid residues and side chain bumps were fixed by the prepared protein structure module in Sybyl-X 2.0 (Tripos International, 2012). Then, the pK values of the residues were predicted and protonized by H++ server (http://biophysics.cs.vt.edu/) (Anandakrishnan et al., 2012). The structure of the mutated peptides was constructed by substituting the sequence of QSF and energy minimized by Sybyl-X 2.0. Then, the molecular dynamics simulations were performed for the complex of PSD95 GK and various peptide binders.

### Molecular Dynamics Simulation

The detailed MD methods were described in our previous work (Shan et al., 2019; Li et al., 2020). In brief, the simulations were performed by the AMBER 2020 packages with ff14SB force field (Maier et al., 2015; Case et al., 2020). Water molecules were treated with the TIP3P water model (Price and Brooks, 2004). The AMBER parameters of phosphorylated serine were obtained from the AMBER parameter database (http://research.bmh.manchester.ac.uk/bryce/amber) (Homeyer et al., 2006). First, three minimization stages were carried out for the systems. Then, each system was gradually heated from 0 to 300 K using the Langevin thermostat during the heating stage and maintained at 300 K during the following equilibrium and production stages.

### Calculation of Binding Free Energies

To calculate the binding free energies of PSD95 GK with the ligands, MD simulations were performed using the aforesaid MD protocol until the systems reached equilibrium. The binding free energies were calculated using the MMPBSA. py implemented in AMBER 2020 (Miller et al., 2012). Totally, 100 snapshots were extracted from the equilibrium trajectory for MM-GBSA free energy calculation. The binding free energy (ΔG_*bind*_) in MM-GBSA between the receptor and ligand is summarized as Equation 1 (Tsui and Case, 2000; Gohlke and Case, 2004). Per-residue energy decomposition was also performed to evaluate the energy contribution of each residue in the systems. All the other parameters were kept as the default value. For per-residue energy decomposition, the binding free energy (ΔG_*bind*_) is divided into three parts as shown in Equation 2, ΔE_*mm*_ denotes the complex gas-phase interaction energy, which comprises van der Waals (ΔE_*vdw*_) and electrostatic (ΔE_*elec*_) interactions and the internal energy variation $\left(\Delta {\text{E}}_{intra}\right)$ (Equation 3). ΔG_*solv*_ represents the difference of the solvation free energy, which is divided into the electrostatic solvation free energy (ΔG_*GB*_) and nonpolar contributions (ΔG_*SA*_). -TΔS represents the contribution of the entropy of the solute molecules. Since the calculation of the entropy is computationally expensive for large systems and tends to introduce low-accuracy approximations, -TΔS is not considered in the present work (Miller et al., 2012).

$$\Delta G_{bind}={\text{G}}_{complex}-\left({\text{G}}_{receptor}+{\text{G}}_{ligand}\right).$$ (1)

$$\Delta G_{bind}=\Delta {\text{E}}_{mm}+\Delta {\text{G}}_{solv}-\text{T}\Delta \text{S}\text{.}$$ (2)

$$\Delta {\text{E}}_{mm}=\Delta {\text{E}}_{vdw}+\Delta {\text{E}}_{elec}+\Delta {\text{E}}_{intra}.$$ (3)

$$\Delta {\text{G}}_{solv}=\Delta {\text{G}}_{GB}+\Delta {\text{G}}_{SA}.$$ (4)

### Protein Expression and Purification

The PSD95 GK domain (a.a. 531-713) was cloned into a modified pET-15b vector with *N*-terminal His6-tag. The construct was expressed in BL21 (DE3) Escherichia coli cells and induced by 0.2 M isopropyl-β-D-thiogalactoside (IPTG) for 18 h at 16°C. His6-tagged protein was first purified using Ni-NTA agarose affinity chromatography (GE Healthcare), and PSD95 GK was then purified by size-exclusion chromatography and then further purified by size-exclusion chromatography (Superdex-200 26/60, GE Healthcare) in the buffer containing 50 mM Tris pH 8.0, 100 mM NaCl, 1 mM EDTA, and 1 mM DTT. The designed peptide inhibitors were commercially synthesized (China Peptides).

### Fluorescence Polarization Assay

Fluorescence polarization assay was performed in 384-well plates (United States Corning) using a SpectraMax Paradigm (Molecular Devices). The FITC-labeled fluorescent peptide (FITC-AARRE (pS)YLKATQ, FITC-SAPAP) and free-labeled peptide (AARRE (pS)YLKATQ) were ordered from China Peptides. The assay buffer contained 50 mM Tris and 100 mM NaCl at pH 7.5. The peptide inhibitors were serially diluted in assay buffer at a top concentration sufficient to yield an 8-point dose–response curve. For each assay, FITC-SAPAP (10 nM) and PSD95 GK protein (2 μM) were added to a final volume of 20 μl in the assay buffer. The polarization values were measured after a 1-h incubation using a SpectraMax Paradigm (Molecular Devices) with excitation and emission wavelengths of 485 and 535 nm, respectively. The FP value was quantified as the difference between the parallel (*I*_ǁ_) and perpendicular (*I*_┴_) emission intensities normalized by the total fluorescence intensity of the emission beam (Equation 5) (Hall et al., 2016). To accomplish the different sensitivity for measuring emission intensities from the *I*_ǁ_ and *I*_┴_ channels, G-factor is often used to correct for any bias toward or against the *I*_┴_ channel (Equation 6). The dissociation constant (*K*_d_) for PSD95 GK/SAPAP was determined using a constant concentration of 10 nM of FITC-SAPAP, and a series of increasing concentrations of PSD95 GK varied from 0 to 20 μM. The *K*_d_ and IC_50_ values were determined from the plot using nonlinear least-squares analysis, and *K*_i_ values were obtained by the formula that is depicted in the article (Nikolovska-Coleska et al., 2004).

$$\text{FP}=\frac{I_{\|}-I_{┴}}{I_{\|}+I_{┴}}.$$ (5)

$$\text{FP}\left(\text{mP}\right)=\;\frac{\left(I_{\|}-I_{┴}\times \text{G}\right)}{\left(I_{\|}+I_{┴}\times \text{G}\right)}\times 1000.$$ (6)

### Microscale Thermophoresis Assay

Microscale thermophoresis (MST) assay was conducted to determine the binding affinity of PSD95 GK and the top peptide F10W using the Monolith NT.115. The His-PSD95 GK protein was kept in the PBS buffer at a concentration of 500 nM and then labeled according to the protocol of the protein labeling kit RED-Tris-NTA. The peptide was diluted in PBS buffer containing 0.05% Tween 20 for the final MST assay. Then, the labeled protein was mixed with the same volume of the diluted peptide (10 μl:10 μl) from different concentrations and incubated for 10 min at 37°C in the dark. Binding was measured by monitoring the thermophoresis with 20% LED power on the Monolith NT.115. The data were analyzed using Mo. Affinity Analysis v2.2.4 software.

### ITC Assay

ITC measurements were carried out on a MicroCal-iTC200 system (Malvern) in a buffer containing 50 mM Tris pH 8.0, 100 mM NaCl, 1 mM EDTA, and 1 mM DTT at 25°C. The concentrations of proteins loaded into the syringe (F10W) and cell (PSD95 GK) were 0.5 and 0.05 mM, respectively. The titration data were analyzed using Origin7.0 from MicalCal and fitted by a one-site binding model.

## Results and Discussion

### Structural Comparison of the Reported PSD95 GK Binders

**FIGURE 1:**
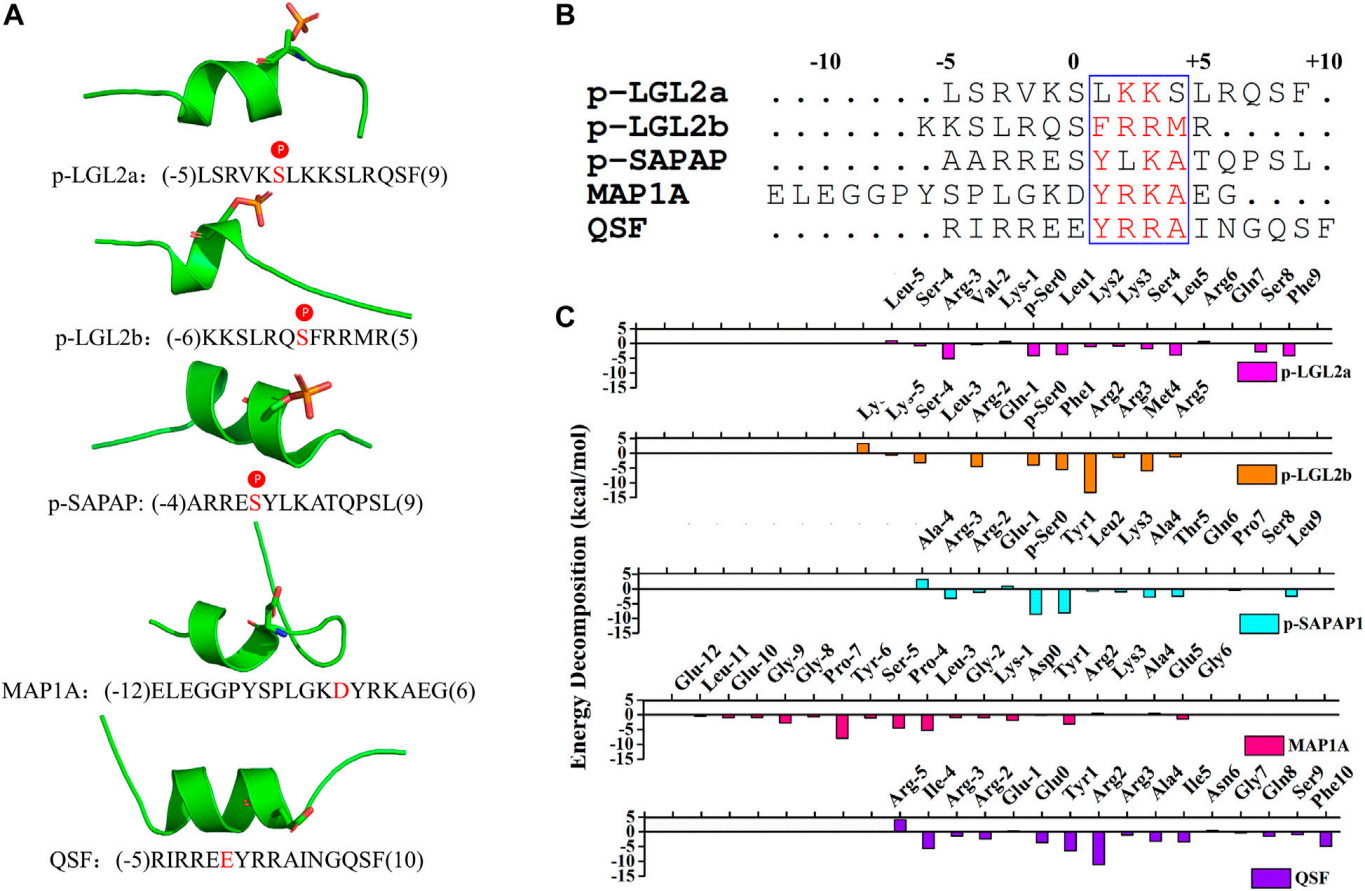
**(A)** Crystal structures of the peptides (p-LGL2a, p-LGL2b, p-SAPAP1, MAP1A, and QSF). The p-Ser or Asp/Glu were displayed in sticks. **(B)** Amino acid sequence alignment of the peptides using Clustal W. Highly conserved residues (conservation score >0.7) were framed in blue according to physicochemical properties. **(C)** Binding energy decomposition of the p-LGL, p-LGL2, p-SAPAP, MAP1A, and QSF peptides.

To investigate the structural features of the peptide binders of PSD95 GK, we accumulated the reported binders and binder affinity data to our knowledge (Supplementary Table S1). These binding partners could be divided into two categories: phosphor-peptides (p-LGL2a, p-LGL2b, p-SAPAP1, and p-LGN) and unphosphorylated binders (MAP1A, MBS domain of KIF13B, QSF, MKL, and DLS). At first, we compared binding affinities between PSD95 GK and its target peptides. As is shown in Supplementary Table S1, phosphor-peptides generally demonstrate more potent binding affinity than unphosphorylated peptides. This is largely due to the substitution of the phosphorylated serine (p-Ser) by Asp or Glu in unphosphorylated peptides which weakened the hydrogen bonding at the phosphor-site formed by R568, R571, and Y609. However, the MBS domain of KIF13B which has 122 residues demonstrates stronger binding affinity than most of the phosphor-peptides resulting from its more extensive binding interface (Zhu et al., 2016). Second, the amino acid sequences of these peptides were further aligned, and the results revealed that the amino acids near the Ser(0) at position 1, 2, 3, and 4 have high amino acid sequence consensus, indicating that they may have the similar binding mode, which is in accordance with the result of our previous study that the GK domain shares a unique recognition mode with the binding partners (Figure 1B) (Li et al., 2020). In addition, the crystal structures of the reported peptides are also displayed in Figure 1A, and it could be found that these peptides all share a short α-helix and two loops at *C*- or *N* -terminal. The key residue p-Ser or Asp/Glu is located at the α-helix region.

### The Binding Interaction of Phosphorylated Peptides

To elucidate the key residues of the binder peptides at the complex interfaces, we first investigated the interactions between the phosphorylated peptides and PSD95 GK by using MD simulations of the reported crystal structures. The structures were generated by CPPTRAJ program in AMBER 2020 for further analysis after the system reached equilibrium state (Roe and Cheatham, 2013). For the PSD95 GK/p-LGL2a complex, at the beginning of the MD simulation, the *N*-terminal loops were exposed toward the solvent and moved freely, which may lead to a rather large fluctuation on the RMSF of 8.87Å (Supplementary Figure 1A). However, when the system reached equilibrium after the 35-ns simulation, the loops moved toward the region between the β3 and β4 sheets of GK forming salt bridges between R (-3) at p-LGL2a and D579_GK_ with the energy contribution of R (-3) to -5.17 kcal/mol, which stabilized the *N*-terminal loops of the p-LGL2a peptide. The salt bridges were also found between the carboxyl group of F10_p-LGL2a_ and the guanidine group of R637_GK_ at α4 helix, which confined the movement of *C*-terminal loops. The hydrophobic residues such as L1 and L5 also possessed crucial hydrophobic interactions which facilitate the assembly of the complex with a binding energy of –3.74 kcal/mol and –3.88 kcal/mol, respectively.

By comparison, GK/p-SAPAP1 and GK/p-LGL2b are more stable than PSD95 GK/p-LGL2b during MD simulation. After analyzing the binding free energy decomposition, we found that the salt bridges were formed between R2 at p-LGL2b and D545 and D549 of GK and also existed between R (-2) at p-LGL2b and S561 and D579 of GK, which both reinforced the binding affinity in the complex of PSD95 GK/p-LGL2b. In addition, the binding was further strengthened by hydrophobic interactions between F1_p-LGL2b_ and M4 _p-LGL2b_ and the residues A601_GK_, G602_GK_, and Y611_GK_. Similarly, for the complex of PSD95 GK/p-SAPAP1, Y1 was found to present the favorite energy contribution of –8.20 kcal/mol, which was inferior to that of p-Ser of −8.60 kcal/mol by forming hydrogen bonds and hydrophobic interactions with T611 from the β6 sheet of the GK domain. The residue R (-3) _p-SAPAP1_ at the loops of *N-*terminal displayed the hydrogen bonds with C562_GK_ and T611 _GK_ and formed salt bridges with D629 _GK_, which confined the movement of the *N*-terminal loops of p-SAPAP1. A4_p-SAPAP1_ possessed the hydrogen bonds and hydrophobic interactions with Q603_GK_, while L9_p-SAPAP1_ possessed the hydrogen bonds and hydrophobic interactions with R676_GK_.

In brief, the phosphor-Ser (pSer0) from each of the three peptides had critical interactions forming a network of hydrogen bonds with R568, Y580, and Y609 of the phosphor-site, and the hydrophobic interactions from the other amino acids were also indispensable for their binding interaction with PSD95 GK.

### The Binding Interaction of Unphosphorylated Peptides

MAP1A and QSF are two unphosphorylated peptides that bind to PSD95 GK. We first characterized these interactions. In the PSD95 GK/MAP1A system, D0_MAP1A_ formed hydrogen bonds with R568_GK_, Y580_GK,_ and Y609_GK_. In addition, hydrogen bonds were also excited between G (-8)_MAP1A_ and D629_GK_, Y (-6)_MAP1A_ and D549_GK_, L (-3)_MAP1A_ and Y580_GK_, and R (2) _MAP1A_ and D549_GK_. Especially, the hydrophobic residues Y (-6), P (-4), and L3 displayed the top three energy contributions with the value of -7.89, -5.27, and -4.45 kcal/mol, which is consistent with the reported experiments that the mutation of hydrophobic residues Y (-6), P (-4), or L3 disrupted the interactions between the peptide and PSD95 GK (Xia et al., 2017).

**FIGURE 2:**
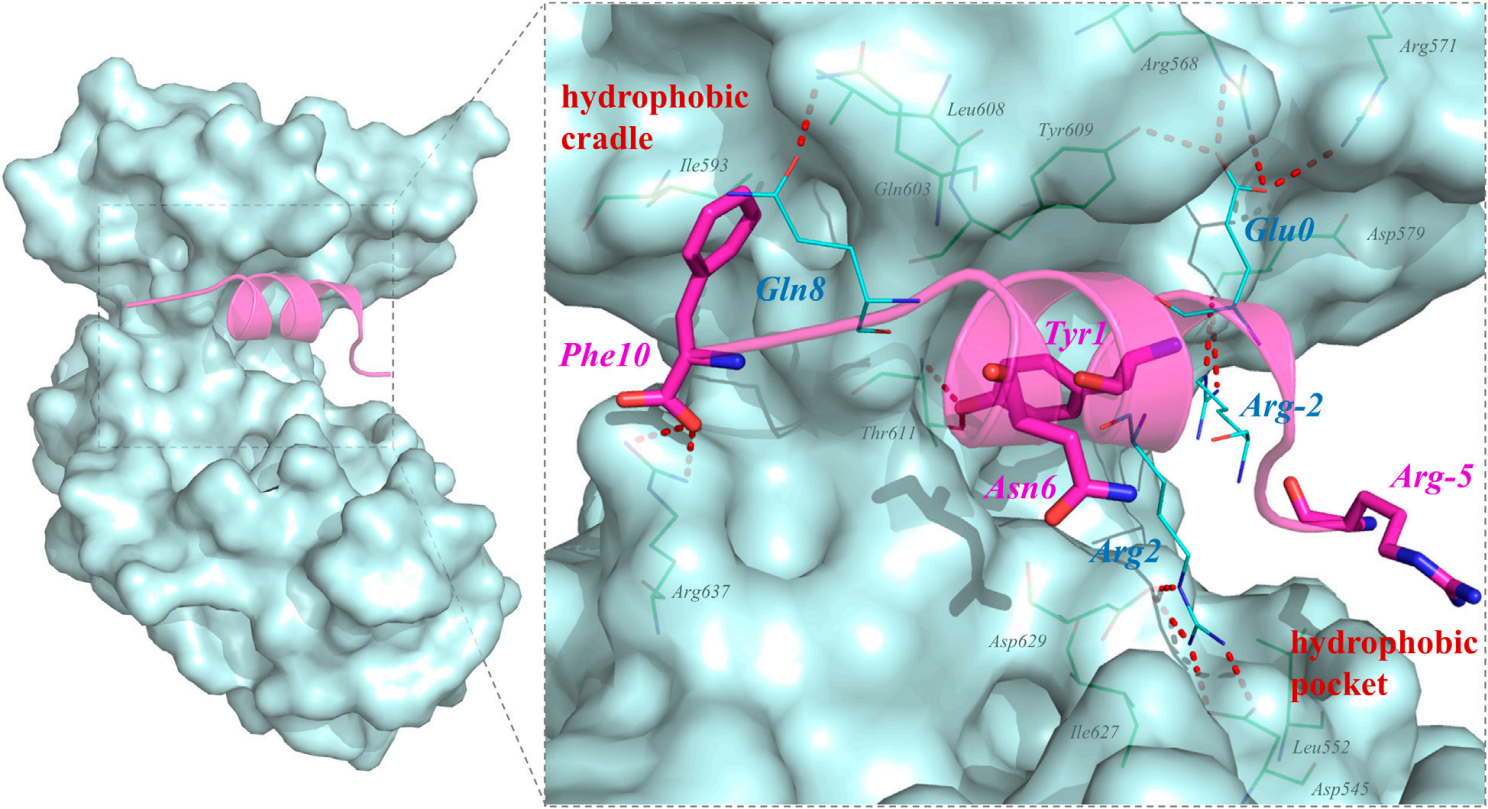
Key residues for the interactions in PSD95 GK/QSF systems during the MD simulations. For the hydrogen bonding interactions, the residues of QSF and GK were displayed in lines and colored by cyan and green, respectively. The residues which were designed to modification were colored by magenta and shown by sticks.

QSF is a synthetic unphosphorylated peptide that integrates the optimal p-SAPAP1 peptide with the *C-*terminal tails of p-LGN or p-LGL2a (Zhu et al., 2017). The interface between PSD95 GK and QSF was also investigated using MD simulation. The results showed that R2_QSF_ has the most favorite energy contribution of -11.06 kcal/mol by forming salt bridges with D545_GK_ and D629_GK_. The following key residues are Y1_QSF_, I (-4)_QSF_, and F (10)_QSF_ which accommodate the hydrophobic site of GK manifesting the hydrophobic interactions with -6.44, -5.59, and -4.88 kcal/mol, respectively. In addition, Y1_QSF_ formed the hydrogen bonds with T611_GK_ which could also be found in PSD95 GK/p-SAPAP1 (Supplementary Figure 3). The residue E (0)_QSF_ formed hydrogen bonds with R568, R571, and Y609 of the GK phosphor-site with −3.66 kcal/mol. The hydrogen bond was found between Q8_QSF_ and Q603_GK_ with −1.46 kcal/mol (Figure 2). While most of the residues possess favorable binding affinity, R (-5) and N6 obviously displayed an unfavorable binding energy contribution of 4.2 kcal/mol and 0.59 kcal/mol, respectively (Figure 1C).

Among unphosphorylated peptides, Asp (MAP1A) or Glu (QSF) mimicking the p-Ser of phosphorylated peptides formed crucial hydrogen bond interactions with R568_GK_, Y580_GK_, and Y609_GK_ of the phosphor-site. Similarly, the hydrophobic interactions were also important for the binding interaction of unphosphorylated peptides.

### Design Strategy of Peptide Inhibitors Using Per-Residue Energy Decomposition

Based on the abovementioned interaction analysis of PSD95 GK and phosphor-/unphosphorylated-peptides, it could be concluded that the binding affinity is mainly dominated by the important hydrogen bonds between p-Ser or Asp/Glu and residues at the phosphor-site of GK, the crucial salt bridges at R (-2)_p-LGL2b_, R (-3)_p-LGL2a, p-SAPAP1_, and R (2) _p-LGL2b,MAP1A,QSF,_ and the indispensable hydrophobic interactions. As for the unphosphorylated QSF peptide possessing relatively low molecular weight and moderate binding affinity, we regarded QSF as the parent peptides for further sequence optimization based on per-residue energy decomposition results.

**FIGURE 3:**
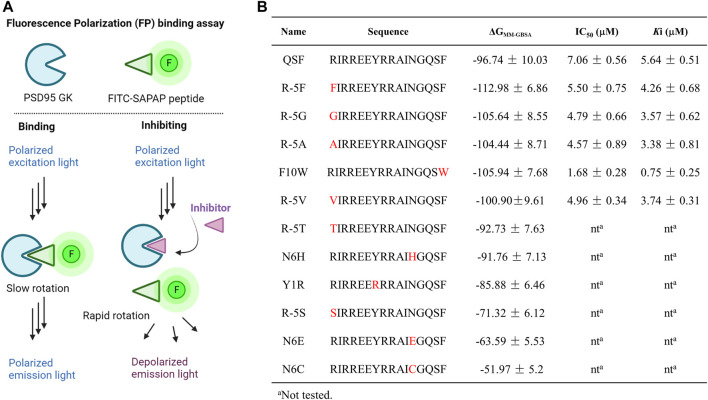
**(A)** Diagrams of the FP assay configurations in the PSD95 GK/FITC-SAPAP system. PSD95 GK binds the FITC-labeled SAPAP peptide, resulting in a high FP signal with polarized emission light; however, competition with inhibitors displaces FITC-SAPAP and reduces the measured polarization with depolarized emission light. **(B)** Binding free energy calculated by the MM-GBSA method and the IC_50_ values were determined by FP assay.

MD simulation and the MM-GBSA method were utilized to calculate the binding free energy of the PSD95 GK/QSF system with a value of −96.74 ± 10.03 kcal/mol. According to the per-residue energy decomposition results, we found that the residue Y1 displayed a crucial binding energy contribution of −6.45 kcal/mol. In order to validate whether the MM-GBSA method was applicable for this study, it was intentionally mutated to arginine to interrupt the hydrophobic and hydrogen bonding interactions. As anticipated, this mutation as Y1R peptide showed a higher binding free energy with a value of −85.88 ± 6.46 kcal/mol than that of QSF (Figure 3B). These results met the theoretical expectation, and it was suggested this approach might be suitable to predict the binding affinity.

Analyzing the distribution of per-residue energy decomposition of PSD95 GK/QSF, R (-5) which is located at the hydrophobic pocket of PSD95 GK, displayed unfavorable energy contribution mainly due to guanidine of R (-5) exposed to the solvent, leading to the relatively high polar solvation energy (Supplementary Table S2). Thus, we modified R (-5) to hydrophobic amino acids as F, G, A, T, S, and V. It can be found that the substitution of hydrophobic amino acids (R-5F, R-5G, R-5A, and R-5V) for arginine at position (-5) increased the binding free energy, while the substitution of hydrophilic amino acids (R-5T, R-5S) decreased the binding interaction, suggesting that (-5) position substitutions were preferred for hydrophobic amino acids.

Similarly, N (6) at α helix displayed slightly unfavorable energy contribution, which was modified as H, E, and C to enhance the hydrophilic interaction. However, the binding free energies of N6H, N6C, and N6E were higher than those of QSF. In addition, the hydrophobic cradle of GK formed by I593, A601, and L608 accommodated the benzene ring of F10, while the hydrophobic space was not fully utilized. The peptide F10W, in which the phenylalanine was replaced by tryptophan, also demonstrated better binding affinity with −105.94 ± 7.68 kcal/mol than QSF (Figure 3B). Therefore, five of the top-ranked peptides with lower binding free energy than QSF were synthetized and evaluated by competitive fluorescence polarization assay.

### F10W Is a Potent GK Inhibitor of PSD95/SAPAP

Fluorescence polarization assay has been widely implemented in high-throughput screening (HTS) and drug discovery (Banco et al., 2018; Miyoshi, 2018; Perveen et al., 2021). The assay could detect the alterations in the apparent molecular weight of a fluorescent probe (or tracer) in solution by observing changes in the polarization of the sample’s emitted light. Therefore, it is usually utilized to interrogate a variety of biological processes, such as a binding event or the enzymatic cleavage of a substrate (Hall et al., 2016). In the competitive FP-based assay, we developed fluorescein isothiocyanate (FITC)–conjugated SAPAP peptide as the fluorescent probe and a His-tagged PSD95 GK domain (His-GK) as the protein. The complex of His-GK with the FITC-SAPAP peptide produced a high FP value that was decreased following the displacement of the labeled peptide by either the unlabeled peptide or inhibitor compounds (Figure 3A). We next chose a low concentration of 10 nM of the probe which could yield a reasonable fluorescent signal and a stable polarization value. To determine the dissociation constant (*K*_d_) for His-GK/FITC-SAPAP, we used a constant concentration of 10 nM of FITC-SAPAP, and a series of increasing concentrations of His-GK varied from 0 to 20 μM. The *K*_d_ of binding between the FITC-SAPAP probe and His-GK was determined to be 4.28 μM, with a dynamic range (ΔmP = mP of bound peptide – mP of free peptide) of 160 ± 5.23 mP. The stability of the FP assay is critical for high-throughput screening, so we incubated the plate at room temperature over a 12-h period. The results showed that the assay is stable and suitable for further high-throughput screening of GK inhibitors, as evidenced by the binding curves (Supplementary Figure S2). Then, the optimal concentration of His-GK was determined to 2 μM with 78 ± 2.34 mP for the competitive binding assay. The competitive binding assay was conducted with 10 nM of FITC-SAPAP, 2 μM of the PSD95 GK domain, and a series of increasing concentrations of the designed peptides. The unlabeled peptide SAPAP was treated as the positive control. The results of competitive fluorescence polarization assay showed that the abovementioned five peptides displayed better inhibitory activity than QSF peptide. Especially, the peptide F10W displayed the most potent inhibitory activity of PSD95/SAPAP with a *K*_i_ value of 0.73 μM (7 times than *K*_i_ value of QSF). To further confirm the binding affinity, the direct interaction between PSD95 GK and F10W was tested by MST and ITC assay with a *K*_d_ value of 0.54 ± 0.21 μM and 0.92 ± 0.45 μM, respectively, which is lower than that of QSF (*K*_d_ = 1.14 ± 0.14 µM in ITC assay).

**FIGURE 4:**
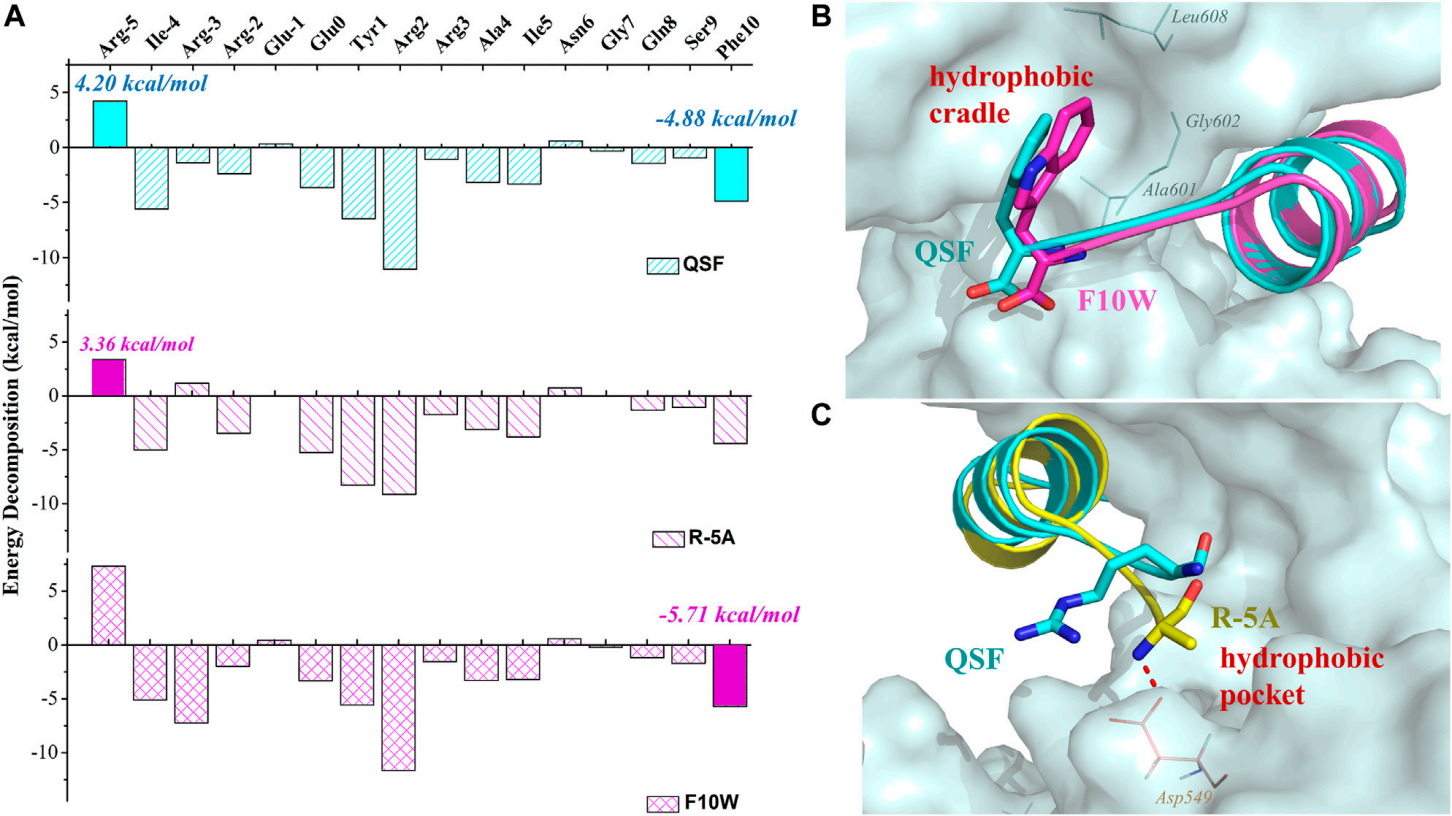
**(A)** Binding free energy decomposition of QSF, R (-5) A, and F10W systems. **(B)** Superposition of the MD equilibrium conformations of QSF and F10W. **(C)** Superposition of the MD equilibrium conformations of QSF and R (-5)A. The hydrogen bond was shown in red dashed line.

To elucidate the difference of inhibitory activity among QSF, F10W, and R5A, the per-residue energy decomposition using the MM-GBSA method was applied. As expected, after the displacement of R (-5) with phenylalanine, glycine, alanine, or valine, the peptide displayed a more favorable energy contribution due to the decreased polar solvation energy (Supplementary Table S5–S8). These hydrophobic residues coupled with I (-4) increased the hydrophobic interactions with the hydrophobic pocket of GK (Figure 4C). In addition, the residue A (-5) of R (-5)A formed the hydrogen bond with D549_GK,_ which reinforced the binding affinity (3.36 kcal/mol in R (-5)A vs. 4.2 kcal/mol in QSF) (Figure 4A). In addition, the energy decomposition of R5V, R5F, and R5G were shown in Supplementary Material (Supplementary Table S6–S8). For F10W, the results showed that tryptophan at (+10) position played a more favorable interaction with the hydrophobic cradle of GK (−5.70 kcal/mol in F10W vs. −4.88 kcal/mol in QSF) (Figures 4A,B). Unexpectedly, the arginine at -3 position of F10W showed obvious improvement on energy contribution compared with QSF (−7.22 kcal/mol in F10W vs. −1.41 kcal/mol in QSF), which was offset by the unfavorable energy contribution of R (-5) with 7.33 kcal/mol. Therefore, we anticipate developing a more potent inhibitory peptide by double-position mutations of F10 and R (-5) in the future research work.

As reported, QSF did not have obvious selectivity for PSD95 GK against DLG1 GK by MD simulation and ITC experiment (Li et al., 2020). These results also suggested that the key residues for the binding of QSF with DLGs are conserved and might not have selectivity for other DLG subfamily members (DLG1-5 GK). Analyzing the binding interactions from the Trp of F10W, we found that the key amino acids (I593, A601, and L608) are identical except for DLG5, suggesting that the modification of QSF to F10W might only affect the selectivity of F10W for DLG5 (Supplementary Figure S6).

## Conclusion

PSD95 belongs to the MAGUK family proteins, a class of proteins that function as molecular scaffolds for the assembly of multiprotein complexes at specialized regions of the plasma membrane (Xu, 2011). PSD95 interacts with SAPAP *via* the GK domain which is crucial for synaptic formation and plasticity and associates with psychiatric disorders (Zhu et al., 2017). In this study, MD simulations were utilized to characterize the binding mechanism between PSD95 GK and five reported peptide binders. The results showed that these peptides bind PSD95 GK in a similar binding pattern. The hydrogen bonds between p-Ser or Asp/Glu and phosphor-site residues, the salt bridges between the negatively charged amino acids and R (-1)_p-LGL_, R (-3)_p-LGL2,p-SAPAP1_, and R (2)_MAP1A,QSF,_ and the hydrophobic interactions are the main driving force for peptides binding to PSD95 GK. Based on the per-residue energy decomposition of the QSF peptide, we designed ten peptides, and the binding affinity was originally evaluated by MD simulations. Then, the competitive FP-based assay for the PSD95 GK/FITC-SAPAP system was developed and utilized to determine the inhibitory activity of five top-ranked peptides. The results demonstrated that F10W is a potent GK inhibitor of PSD95/SAPAP, mostly due to its well accommodation with the hydrophobic cradle of GK. In addition, the substitution of R (-5) with hydrophobic residues could also improve the binding affinity by increasing the hydrophobic interactions with the hydrophobic pocket of GK. Taken together, the results obtained in this study may be valuable for designing potent peptide inhibitors, and the strategy of enhancing the hydrophobic interactions is an important method for the design of new peptides targeting PSD95 GK.

## Data Availability

The original contributions presented in the study are included in the article/Supplementary Material, further inquiries can be directed to the corresponding authors.
