# Supplementary material for: Application of per-Residue Energy Decomposition to Design Peptide Inhibitors of PSD95 GK Domain
Source: Front Mol Biosci. 2022 Mar 30;9:848353. doi: 10.3389/fmolb.2022.848353 (PMC9005747; doi:10.3389/fmolb.2022.848353)
Supplement: Supplementary file 1 [file DataSheet1.PDF]

Supplementary Material

Application of per-residue energy decomposition to design peptide inhibitors of PSD95 GK domain

Miao Tian[1#], Hongwei Li[2#], Xiao Yan[2], Jing Gu[2], Pengfei Zheng[2], Sulan Luo[1], Dongting Zhangsun[1*], Qiong Chen[3*], Qin Ouyang[2*]

[1] Key Laboratory of Tropical Biological Resources of Ministry of Education, School of Pharmaceutical Sciences, Hainan University, Haikou, China.

[2] Department of Pharmaceutical Chemistry, Third Military Medical University, Chongqing, China

[3] Department of Neurology, Xinqiao Hospital, Third Military Medical University, Chongqing, China

[#]These authors contributed equally.

Email:

Dongting Zhangsun, Zhangsundt@163.com; Qiong Chen, besefeniqq@163.com; Qin Ouyang, ouyangq@tmmu.edu.cn

1. Supplementary Figures

Supplementary Figure 1 The RMSD and RMSF of the PSD95 GK and p-LGL2a (A), p-LGL2b (B), p-SAPAP1 (C), MAP1A (D) and QSF (E).

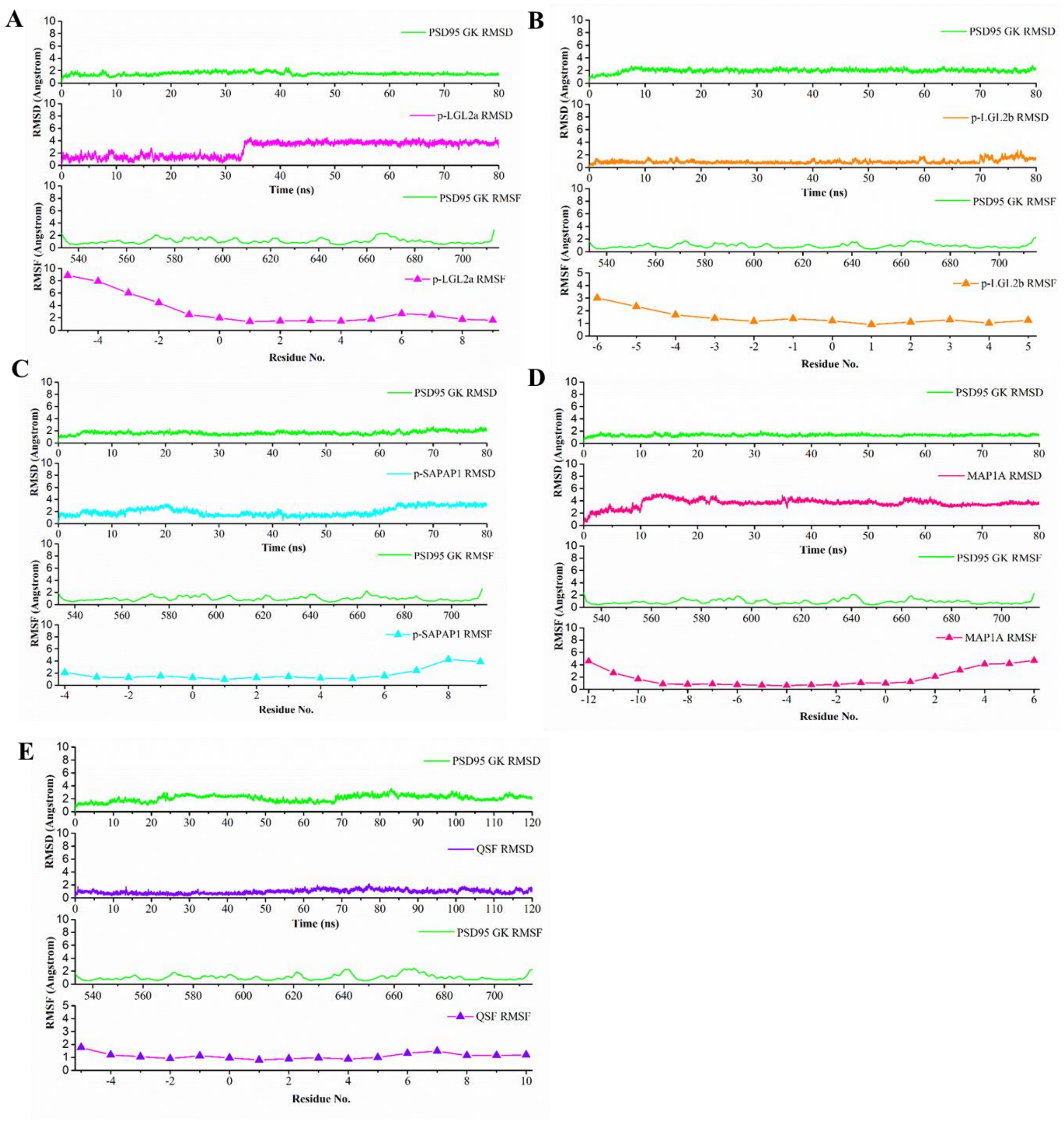

Supplementary Figure 2 Stability of binding experiments between the FITC-SAPAP probe and His-GK over 12 hours.

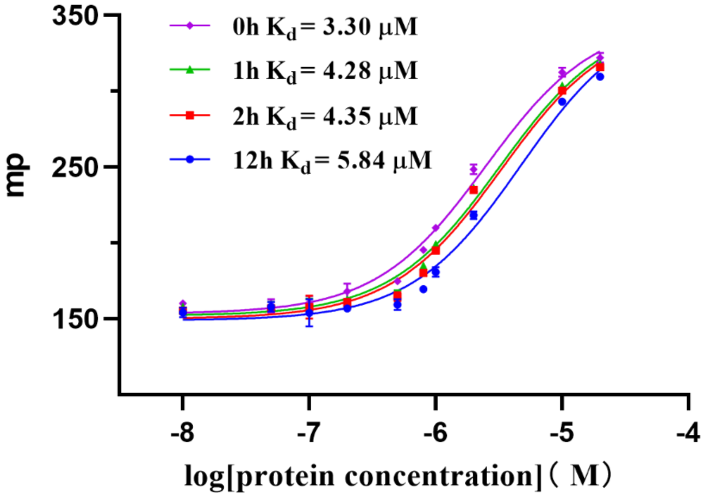

Supplementary Figure 3 The key residues for the interactions in PSD95 GK/QSF in 2D.

QSF: (-5)RIRREEYRRAINGQSF(10)

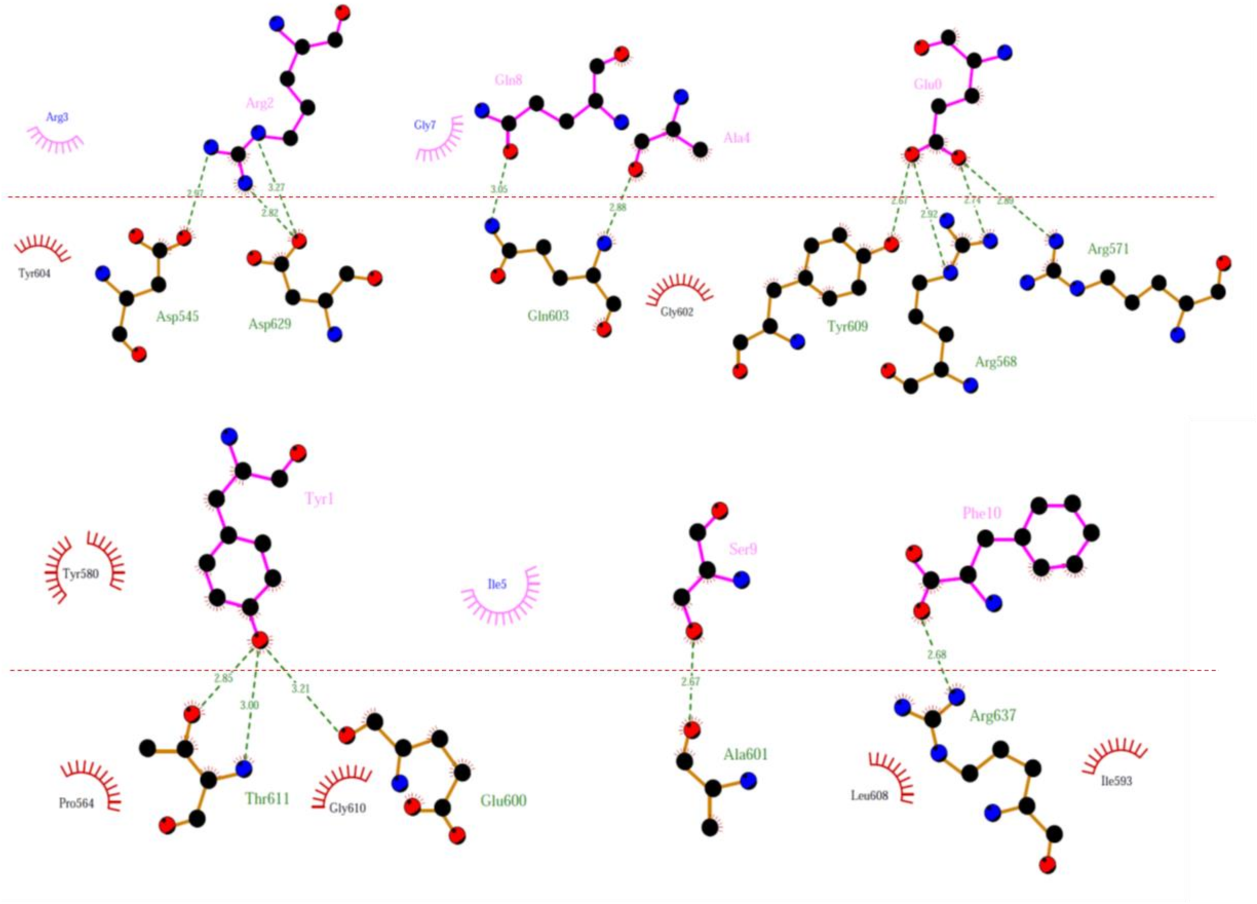

Supplementary Figure 4 The ITC-based measurement of the binding affinity of PSD95 GK/F10W interactions

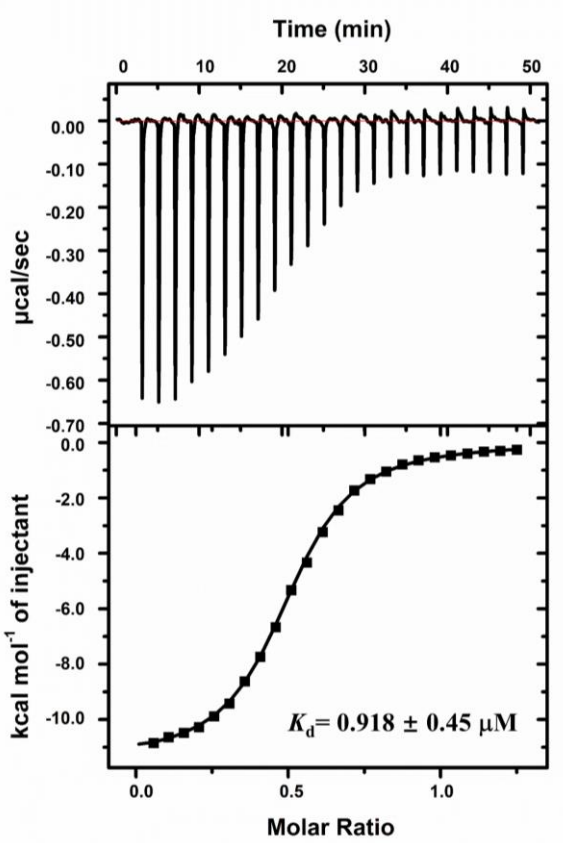

Supplementary Figure 5 The MST-based measurement of the binding affinity of PSD95 GK/F10W interactions

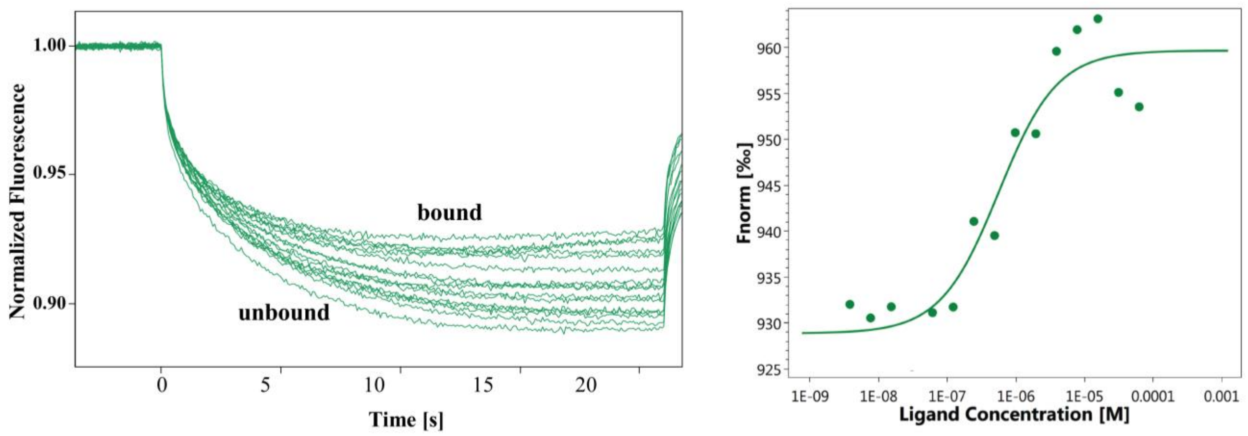

Supplementary Figure 6 The sequence alignment of the DLGs GK domains. Highly conserved residues (conservation score > 0.7) were framed in blue according to physico-chemical properties.

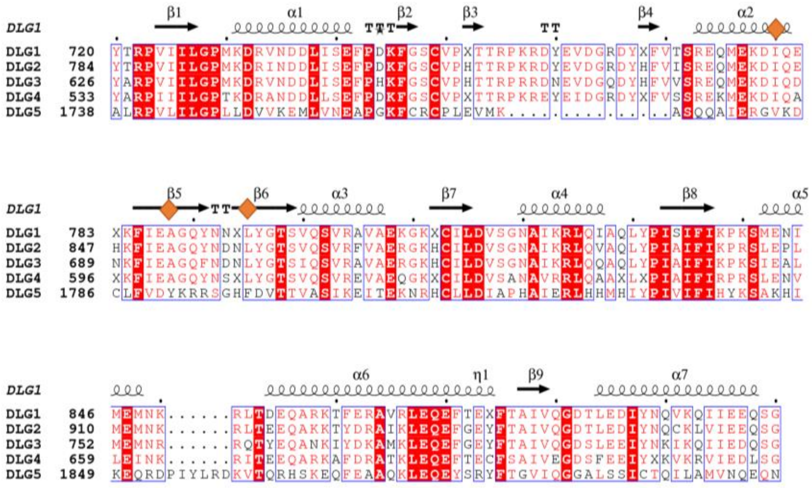

2. Supplementary Tables

Supplementary Table 1. The reported GK domain binders of PSD95

Binding partner	Amino acid sequences	K_d value (μM)	References
p-LGL	KKSLRQ(pS)FRRMR	N/M	Zhu et al., 2014
p-LGL2	LSRVK(pS)LKKSLRQSF	10.2 ± 1.3[a]	Zhu et al., 2014
p-SAPAP	AARRE(pS)YLKATQPSL	0.08 ± 0.01[b]	Zhu et al., 2017
p-LGN	GRRH(pS)MENLELMKLTPEK	0.53 ± 0.09[a]	Zhu et al., 2011
MAP1A	AELEGGPYSPLGKDYRKAEGEREEEG	12.1 ± 0.2[a]	Xia et al., 2017
KIF13B	MBS domain	0.07 ± 0.00[b]	Zhu et al., 2016
QSF(synthetic peptide)	RIRREEYRRAINGQSF	1.14 ± 0.14[b]	Zhu et al., 2017
MKL(synthetic peptide)	RIRREEYRRAINGQSMKL	1.86 ± 0.31[b]	Zhu et al., 2017
DLS(synthetic peptide)	RIRREEYRRAINGQSFDLS	0.66 ± 0.13[b]	Zhu et al., 2017
Stapled peptide (synthetic peptide)	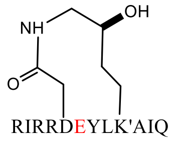	1.36 ± 0.05[b]	I. C.et al., 2021

N/M: not mentioned, [a]measured by fluorescence polarization assay, [b]measured by isothermal titration calorimetry assay.

Supplementary Table 2. The binding free energy calculated by MM-GBSA method.

Residue	van der Waals	Electrostatic	Polar Solvation	Non-Polar Solv.	TOTAL
QSF	-78.54±6.26	-621.25±59.09	616.45±52.92	-13.40±0.59	-96.74±10.03
R-5F	-84.70±5.15	-649.01±24.63	635.10±21.69	-14.37±0.37	-112.98±6.86
R-5G	-79.61±6.30	-642.85±33.77	630.78±31.47	-13.96±0.41	-105.64±8.55
R-5A	-85.13±5.73	-659.71 ± 51.70	654.97± 49.01	-14.57±0.58	-104.44±8.71
F10W	-80.42±5.47	-694.45±30.10	682.85±25.56	-13.92±0.50	-105.94±7.68
R-5V	-84.47±6.48	-560.36±33.41	557.81±25.20	-13.88±0.41	-100.90±9.61
R-5T	-86.24± 32.42	-530.59±32.42	538.17±28.57	-14.06±0.49	-92.73±7.63
N6H	-104.07±5.54	-643.22±29.79	671.64±26.00	-16.10 ± 0.57	-91.76±7.13
Y1R	-83.17±5.87	-663.89±41.83	675.52±37.92	-14.34±0.46	-85.88±6.46
R-5S	-85.47±3.90	-501.80±24.64	529.15±22.48	-13.21±0.39	-71.32±6.12
N6E	-85.27±5.48	-306.13±37.30	339.77±34.42	-11.97±0. 50	-63.59±5.53
N6C	-67.04±4.17	-456.32±24.95	481.78±21.81	-10.38±0.44	-51.97±5.2

All the energies are in kcal/mol.

Supplementary Table 3. The binding free energy decomposition of the residues in QSF

Residue	van der Waals	Electrostatic	Polar Solvation	Non-Polar Solv.	TOTAL
Arg-5	-1.05 ± 0.95	-84.27 ± 18.24	89.78 ± 16.67	-0.25 ± 0.19	4.2 ± 2
Ile-4	-5.27 ± 0.74	-2.87 ± 2.11	3.63 ± 1.46	-1.09 ± 0.07	-5.6 ± 1.01
Arg-3	-1.88 ± 0.88	-34.66 ± 12.16	35.38 ± 11.36	-0.25 ± 0.18	-1.41 ± 1.88
Arg-2	-4.24 ± 0.75	-39.96 ± 5.55	42.62 ± 5.02	-0.8 ± 0.08	-2.38 ± 1.38
Glu-1	-0.42 ± 0.13	15.41 ± 5.17	-14.67 ± 5.23	0 ± 0.01	0.32 ± 0.31
Glu0	-1.86 ± 1.23	-47.77 ± 8.16	46.78 ± 6.82	-0.81 ± 0.11	-3.66 ± 1.99
Tyr1	-6.86 ± 0.87	-6.61 ± 1.57	8.01 ± 1.22	-0.99 ± 0.05	-6.45 ± 1.23
Arg2	0.46 ± 1.25	-64.5 ± 7.01	53.48 ± 5.23	-0.5 ± 0.07	-11.06 ± 2.36
Arg3	-2.8 ± 0.6	7.53 ± 3.51	-5.41 ± 3.23	-0.4 ± 0.08	-1.08 ± 0.63
Ala4	-3.49 ± 0.45	-2.03 ± 0.78	2.78 ± 0.56	-0.43 ± 0.03	-3.17 ± 0.59
Ile5	-3.34 ± 0.64	-2.48 ± 0.46	2.84 ± 0.46	-0.36 ± 0.08	-3.34 ± 0.65
Asn6	-0.35 ± 0.06	-1.77 ± 1.46	2.71 ± 1.52	0 ± 0.02	0.59 ± 0.16
Gly7	-0.62 ± 0.21	-0.86 ± 0.44	1.21 ± 0.4	-0.07 ± 0.04	-0.34 ± 0.36
Gln8	-2.04 ± 0.64	-5.02 ± 2.64	5.89 ± 1.99	-0.29 ± 0.06	-1.46 ± 0.86
Ser9	-1.16 ± 0.54	-2.79 ± 1.91	3.07 ± 1.39	-0.08 ± 0.06	-0.95 ± 0.77
Phe10	-4.36 ± 1.05	-37.97 ± 7.54	38.6 ± 6.6	-1.16 ± 0.11	-4.88 ± 1.38

All the energies are in kcal/mol.

Supplementary Table 4. The binding free energy decomposition of the residues in F10W

Residue	van der Waals	Electrostatic	Polar Solvation	Non-Polar Solv.	TOTAL
Arg-5	-1.47 ± 0.54	-62.76 ± 6.57	71.8 ± 5.74	-0.24 ± 0.19	7.33 ± 1.34
Ile-4	-5.6 ± 0.57	-1.83 ± 0.75	3.42 ± 0.7	-1.07 ± 0.05	-5.09 ± 0.6
Arg-3	-2.38 ± 0.86	-75.45 ± 3.52	71.15 ± 2.88	-0.54 ± 0.04	-7.23 ± 1.13
Arg-2	-4.12 ± 0.67	-35.7 ± 3.97	38.59 ± 3.59	-0.74 ± 0.06	-1.98 ± 1.12
Glu-1	-0.35 ± 0.04	8.55 ± 3.7	-7.78 ± 3.63	0 ± 0	0.42 ± 0.13
Glu0	-1.89 ± 1.34	-48.54 ± 4.94	47.93 ± 4.14	-0.81 ± 0.07	-3.32 ± 1.51
Tyr1	-6.07 ± 0.72	-6.77 ± 1.22	8.2 ± 0.95	-0.92 ± 0.05	-5.56 ± 0.81
Arg2	0.19 ± 0.91	-69.8 ± 4.39	58.47 ± 3.1	-0.52 ± 0.04	-11.66 ± 1.77
Arg3	-2.4 ± 0.35	2.16 ± 2.06	-0.95 ± 1.96	-0.37 ± 0.03	-1.56 ± 0.65
Ala4	-3.48 ± 0.51	-2.49 ± 0.67	3.11 ± 0.45	-0.44 ± 0.02	-3.3 ± 0.54
Ile5	-3.17 ± 0.43	-2.58 ± 0.31	2.9 ± 0.31	-0.36 ± 0.06	-3.2 ± 0.49
Asn6	-0.37 ± 0.04	-1.51 ± 1.49	2.48 ± 1.5	0 ± 0	0.59 ± 0.1
Gly7	-0.68 ± 0.12	0.15 ± 0.4	0.37 ± 0.32	-0.06 ± 0.02	-0.22 ± 0.19
Gln8	-1.99 ± 0.52	-3.02 ± 1.36	4.09 ± 1.41	-0.26 ± 0.05	-1.18 ± 0.53
Ser9	-1 ± 0.69	-3.48 ± 1.41	2.83 ± 0.76	-0.04 ± 0.03	-1.7 ± 0.68
Trp10	-5.43 ± 0.94	-44.15 ± 4.02	45.13 ± 3.96	-1.26 ± 0.09	-5.71 ± 1.18

All the energies are in kcal/mol.

Supplementary Table 5. The binding free energy decomposition of the residues in R(-5)A

Residue	van der Waals	Electrostatic	Polar Solvation	Non-Polar Solv.	TOTAL
Ala-5	-0.67 ± 0.59	-57.09 ± 16.49	61.35 ± 15.12	-0.23 ± 0.1	3.36 ± 2.1
Ile-4	-4.3 ± 0.85	-2.06 ± 2.45	2.23 ± 1.64	-0.86 ± 0.1	-4.99 ± 1.45
Arg-3	-0.69 ± 0.09	-18.22 ± 4.44	20.12 ± 4.42	-0.02 ± 0.02	1.19 ± 0.28
Arg-2	-4.74 ± 0.77	-47.63 ± 5.91	49.82 ± 5.54	-0.92 ± 0.05	-3.48 ± 1.06
Glu-1	-0.62 ± 0.09	2.25 ± 3.44	-1.65 ± 3.43	0 ± 0	-0.03 ± 0.15
Glu0	-3.27 ± 1.11	-48.18 ± 8.8	47.1 ± 7.13	-0.91 ± 0.07	-5.26 ± 2.57
Tyr1	-6.98 ± 0.74	-11.33 ± 1.51	11.03 ± 0.73	-1 ± 0.04	-8.28 ± 0.95
Arg2	-0.44 ± 1.02	-56.48 ± 6.23	48.34 ± 5.32	-0.57 ± 0.08	-9.14 ± 1.61
Arg3	-2.45 ± 0.48	4.65 ± 3.2	-3.56 ± 3.01	-0.37 ± 0.03	-1.72 ± 0.59
Ala4	-3.39 ± 0.55	-2.13 ± 0.56	2.83 ± 0.56	-0.42 ± 0.04	-3.1 ± 0.59
Ile5	-3.7 ± 0.56	-2.42 ± 0.42	2.79 ± 0.43	-0.46 ± 0.06	-3.79 ± 0.62
Asn6	-0.4 ± 0.07	-1.77 ± 1.8	2.9 ± 1.85	-0.02 ± 0.05	0.72 ± 0.22
Gly7	-0.46 ± 0.17	-0.41 ± 0.42	0.91 ± 0.37	-0.03 ± 0.02	0.02 ± 0.25
Gln8	-1.94 ± 0.66	-5.15 ± 3.17	6.09 ± 2.54	-0.31 ± 0.09	-1.31 ± 1
Ser9	-1.09 ± 0.51	-3.38 ± 2.14	3.5 ± 1.44	-0.09 ± 0.07	-1.06 ± 0.84
Phe10	-4.33 ± 1.15	-36.24 ± 10.17	37.29 ± 8.76	-1.12 ± 0.14	-4.41 ± 1.57

All the energies are in kcal/mol.

Supplementary Table 6. The binding free energy decomposition of the residues in R(-5)V

Residue	van der Waals	Electrostatic	Polar Solvation	Non-Polar Solv.	TOTAL
Val-5	-1.99 ± 0.84	67.9 ± 4.71	71.22 ± 4.78	-0.57 ± 0.08	0.77 ± 0.92
Ile-4	-5.87 ± 0.84	-6.02 ± 1.03	5.33 ± 0.75	-1.03 ± 0.04	-7.59 ± 0.57
Arg-3	-1.13 ± 0.19	-18.98 ± 3.43	20.67 ± 3.43	-0.05 ± 0.03	0.5 ± 0.2
Arg-2	-4.52 ± 0.58	-34.92 ± 3.17	37.51 ± 2.95	-0.91 ± 0.04	-2.84 ± 0.99
Glu-1	-0.62 ± 0.11	5.85 ± 2.72	-5.27 ± 2.72	0 ± 0	-0.04 ± 0.18
Glu0	-3.5 ± 0.87	-28.77 ± 10.03	29.37 ± 8.61	-0.77 ± 0.09	-3.67 ± 1.77
Tyr1	-6.78 ± 0.92	-6.59 ± 1.59	8.1 ± 0.86	-1.01 ± 0.05	-6.27 ± 1.18
Arg2	0.81 ± 1.26	-71.64 ± 4.94	57.62 ± 3.76	-0.44 ± 0.03	-13.65 ± 1.49
Arg3	-2.52 ± 0.51	2.98 ± 2.66	-1.67 ± 2.44	-0.36 ± 0.04	-1.56 ± 0.53
Ala4	-3.43 ± 0.4	-1.58 ± 0.8	2.28 ± 0.58	-0.42 ± 0.03	-3.16 ± 0.51
Ile5	-3.88 ± 0.62	-1.99 ± 0.4	2.38 ± 0.36	-0.38 ± 0.06	-3.88 ± 0.65
Asn6	-0.41 ± 0.08	-1.63 ± 1.64	2.64 ± 1.61	-0.04 ± 0.05	0.57 ± 0.18
Gly7	-0.47 ± 0.15	-0.4 ± 0.28	0.84 ± 0.24	-0.04 ± 0.02	-0.08 ± 0.21
Gln8	-2.16 ± 0.68	-5.23 ± 2.67	6.47 ± 1.6	-0.31 ± 0.04	-1.23 ± 0.94
Ser9	-1 ± 0.54	-3.17 ± 2.07	3.02 ± 1.34	-0.1 ± 0.04	-1.25 ± 0.72
Phe10	-4.77 ± 0.78	-40.2 ± 4.81	40.89 ± 4.28	-1.16 ± 0.08	-5.24 ± 1.08

All the energies are in kcal/mol.

Supplementary Table 7. The binding free energy decomposition of the residues in R(-5)F

Residue	van der Waals	Electrostatic	Polar Solvation	Non-Polar Solv.	TOTAL
Phe-5	-2.16 ± 0.41	-39.52 ± 4.79	48.46 ± 5.37	-0.44 ± 0.1	6.34 ± 1.33
Ile-4	-5.18 ± 0.42	-2.09 ± 0.52	3 ± 0.51	-0.9 ± 0.04	-5.16 ± 0.44
Arg-3	-2.39 ± 0.91	-69.04 ± 4.14	65.23 ± 3.54	-0.55 ± 0.03	-6.74 ± 1.21
Arg-2	-4.77 ± 0.72	-35.43 ± 3.32	37.71 ± 3.27	-0.86 ± 0.05	-3.36 ± 0.9
Glu-1	-0.65 ± 0.07	4.23 ± 3.1	-3.29 ± 3.07	-0.01 ± 0.01	0.28 ± 0.15
Glu0	-2.54 ± 1.2	-61.19 ± 4.99	58.45 ± 3.81	-0.92 ± 0.04	-6.2 ± 1.75
Tyr1	-7.44 ± 0.78	-5.78 ± 1.14	6.84 ± 0.78	-1 ± 0.03	-7.39 ± 0.83
Arg2	0.28 ± 1.22	-61.43 ± 5.26	51.45 ± 3.57	-0.53 ± 0.02	-10.23 ± 1.7
Arg3	-2.44 ± 0.44	5.59 ± 2.53	-4.46 ± 2.38	-0.35 ± 0.04	-1.66 ± 0.6
Ala4	-3.36 ± 0.44	-1.62 ± 0.61	2.5 ± 0.58	-0.41 ± 0.02	-2.89 ± 0.45
Ile5	-2.8 ± 0.38	-2.3 ± 0.41	2.54 ± 0.37	-0.3 ± 0.05	-2.86 ± 0.43
Asn6	-0.37 ± 0.06	-2.33 ± 1.28	3.35 ± 1.33	-0.02 ± 0.03	0.64 ± 0.14
Gly7	-0.41 ± 0.16	-0.5 ± 0.32	0.89 ± 0.24	-0.04 ± 0.03	-0.05 ± 0.21
Gln8	-1.93 ± 0.54	-6.48 ± 2.24	7.14 ± 1.45	-0.3 ± 0.04	-1.58 ± 0.79
Ser9	-1.42 ± 0.29	-2.14 ± 1.65	2.79 ± 1.24	-0.09 ± 0.04	-0.87 ± 0.57
Phe10	-4.76 ± 1.01	-44.46 ± 5.78	45.18 ± 4.41	-1.17 ± 0.08	-5.21 ± 1.35

All the energies are in kcal/mol.

Supplementary Table 8. The binding free energy decomposition of the residues in R(-5)G

Residue	van der Waals	Electrostatic	Polar Solvation	Non-Polar Solv.	TOTAL
Gly-5	-0.51 ± 0.22	-37.31 ± 5.1	41.93 ± 5.3	-0.06 ± 0.07	4.05 ± 0.63
Ile-4	-4.99 ± 0.73	-2.43 ± 0.71	3.86 ± 0.71	-1.15 ± 0.08	-4.72 ± 0.71
Arg-3	-2.23 ± 1.1	-76.33 ± 4.92	71.72 ± 4.52	-0.62 ± 0.08	-7.46 ± 1.15
Arg-2	-4.41 ± 0.78	-37.03 ± 5.38	39.83 ± 4.8	-0.88 ± 0.06	-2.48 ± 1.08
Glu-1	-0.57 ± 0.09	4.8 ± 3.9	-4.05 ± 3.84	0 ± 0	0.17 ± 0.15
Glu0	-2.69 ± 1.37	-49.21 ± 5.7	47.42 ± 4.34	-0.85 ± 0.05	-5.33 ± 2.18
Tyr1	-7.33 ± 0.72	-6.45 ± 1.22	7.67 ± 0.92	-1.04 ± 0.04	-7.15 ± 0.89
Arg2	0.41 ± 1.56	-68.18 ± 3.92	58.02 ± 2.91	-0.6 ± 0.04	-10.34 ± 1.52
Arg3	-2.46 ± 0.53	3.17 ± 3.59	-1.68 ± 3.26	-0.38 ± 0.04	-1.34 ± 0.78
Ala4	-3.5 ± 0.42	-2.45 ± 0.78	3.08 ± 0.54	-0.42 ± 0.03	-3.28 ± 0.65
Ile5	-2.98 ± 0.33	-2.98 ± 0.44	3.32 ± 0.45	-0.3 ± 0.05	-2.94 ± 0.37
Asn6	-0.36 ± 0.04	-2.17 ± 1.75	3.17 ± 1.79	0 ± 0	0.64 ± 0.11
Gly7	-0.65 ± 0.18	-0.86 ± 0.7	1.22 ± 0.64	-0.07 ± 0.03	-0.36 ± 0.29
Gln8	-2.12 ± 0.62	-4.41 ± 2.12	5.73 ± 1.59	-0.3 ± 0.05	-1.09 ± 0.94
Ser9	-1.09 ± 0.51	-3.59 ± 1.66	3.65 ± 1.18	-0.09 ± 0.05	-1.11 ± 0.87
Phe10	-4.32 ± 0.8	-36.04 ± 6.48	36.84 ± 5.34	-1.14 ± 0.08	-4.66 ± 1.31

All the energies are in kcal/mol.

Reference:

Zhu, J., Shang, Y., Wan, Q., Xia, Y., Chen, J., Du, Q., et al. (2014). Phosphorylation-dependent interaction between tumor suppressors Dlg and Lgl. *Cell Res* 24(4)**,** 451-463. doi: 10.1038/cr.2014.16.

Zhu, J., Zhou, Q., Shang, Y., Li, H., Peng, M., Ke, X., et al. (2017). Synaptic Targeting and Function of SAPAPs Mediated by Phosphorylation-Dependent Binding to PSD-95 MAGUKs. *Cell Rep* 21(13)**,** 3781-3793. doi: 10.1016/j.celrep.2017.11.107.

Zhu, J., Shang, Y., Xia, C., Wang, W., Wen, W., and Zhang, M. (2011). Guanylate kinase domains of the MAGUK family scaffold proteins as specific phospho-protein-binding modules. *EMBO J* 30(24)**,** 4986-4997. doi: 10.1038/emboj.2011.428.

Xia, Y., Shang, Y., Zhang, R., and Zhu, J. (2017). Structure of the PSD-95/MAP1A complex reveals a unique target recognition mode of the MAGUK GK domain. *Biochem J* 474(16)**,** 2817-2828. doi: 10.1042/BCJ20170356.

Zhu, J., Shang, Y., Xia, Y., Zhang, R., and Zhang, M. (2016). An Atypical MAGUK GK Target Recognition Mode Revealed by the Interaction between DLG and KIF13B. *Structure* 24(11)**,** 1876-1885. doi: 10.1016/j.str.2016.08.008.

Unarta, I.C., Xu, J., Shang, Y., Cheung, C.H.P., Zhu, R., Chen, X., et al. (2021). Entropy of stapled peptide inhibitors in free state is the major contributor to the improvement of binding affinity with the GK domain. *RSC Chem Biol* 2(4)**,** 1274-1284. doi: 10.1039/d1cb00087j.
